# Effects of Habitual Dietary Change on the Gut Microbiota and Health of Silkworms

**DOI:** 10.3390/ijms25031722

**Published:** 2024-01-31

**Authors:** Guang Wang, Xueyan Ding, Jiameng Yang, Lu Ma, Xiaoning Sun, Ruihong Zhu, Riming Lu, Zhitian Xiao, Zhiyi Xing, Jingbin Liu, Zhonghua Pan, Shiqing Xu, Yanghu Sima

**Affiliations:** 1 School of Biology and Basic Medical Sciences, Suzhou Medical College, Soochow University, Suzhou 215123, China; gwang1994@suda.edu.cn (G.W.); szsqxu@suda.edu.cn (S.X.); 2 Institute of Agricultural Biotechnology & Ecology (IABE), Soochow University, Suzhou 215123, China

**Keywords:** *Bombyx mori*, gut microbiota, dietary transition, immune resistance, cocoon silk production

## Abstract

Diet plays a crucial role in shaping the gut microbiota and overall health of animals. Traditionally, silkworms are fed fresh mulberry leaves, and artificial diets do not support good health. The aim of this study was to explore the relationship between the dietary transition from artificial diets to mulberry leaves and the effects on the gut microbiota and physiological changes in silkworms as a model organism. With the transition from artificial diets to mulberry leaves, the diversity of the silkworm gut microbiota increased, and the proportion of *Enterococcus* and *Weissella*, the dominant gut bacterial species in silkworms reared on artificial diets, decreased, whereas the abundance of *Achromobacter* and *Rhodococcus* increased. Dietary transition at different times, including the third or fifth instar larval stages, resulted in significant differences in the growth and development, immune resistance, and silk production capacity of silkworms. These changes might have been associated with the rapid adaptation of the intestinal microbiota of silkworms to dietary transition. This study preliminarily established a dietary transition–gut microbial model in silkworms based on the conversion from artificial diets to mulberry leaves, thus providing an important reference for future studies on the mechanisms through which habitual dietary changes affect host physiology through the gut microbiome.

## 1. Introduction

Dietary patterns are considered the most important factor shaping the gut microbiota [1,2]. Similar to mammals, the gut microbiota of silkworms also plays a crucial role in the host’s nutritional metabolism and immune defense [3,4,5]. In insect models such as *Bombyx mori* and *Drosophila*, as well as in mammalian models such as mice, dietary changes have been shown to affect the plasticity of gut microbiota and to be closely associated with health [6,7,8,9]. The use of diet-induced microbiome changes to optimize host health has received increasing attention from the scientific community [10,11,12].

Over thousands of years of evolution, silkworms have developed a habit of oligophagy of fresh mulberry leaves (MLs), which are considered the perfect nutritional source for these organisms [13,14]. In order to improve production efficiency, modern sericulture has gradually started using artificial diets (ADs) as a substitute for MLs in silkworm rearing [15]. However, silkworms fed on ADs exhibit significantly diminished robustness and silk production efficiency [16,17]. Studies using mice and other models have revealed that a high-fat diet (HFD) or high-sugar diet (HSD) significantly affects the composition and function of the gut microbiota, thus impairing the host’s metabolic homeostasis [18,19]. The microbiome imbalance caused by the shift from a low-fat and high-fiber diet to an HFD or HSD is the basis of this intestinal microecological imbalance [20,21]. A high-fiber diet has been shown to effectively alleviate lipid and carbohydrate metabolism disorders caused by HFD and HSD by optimizing the gut microbiota and increasing microbiome-encoded glycan-degrading carbohydrate-active enzymes [22,23], thereby improving health conditions [24,25]. Although these research advances have confirmed the effects of dietary interventions in reshaping the gut microbiota, few studies have assessed the effects of dietary interventions at different stages of the life cycle on the gut microbiota and host health [10,26]. Therefore, we sought to explore the relationship between the period of dietary transition and the reshaping of the gut microbiota.

*B. mori* is an ideal model for studying the interaction between gut microbiota and the host [27,28,29]. Our previous research has indicated that, under feeding on MLs, different silkworm individuals showed highly similar gut microbiota. However, when AD replaced ML feeding in silkworms, the gut microbiota composition and abundance were markedly altered [30]. Therefore, we replaced ADs with MLs at different times during the larval stage to establish a silkworm model of the dietary transition–gut microbiota. We subsequently studied the relationship between dietary transitions at different stages and health in silkworms and identified the roles of gut microbiota changes in this process. Our research findings provide a reference for further studies aimed at elucidating the mechanisms through which dietary interventions reshape the gut microbiota and influence host health.

## 2. Results

### 2.1. Comparison of the Gut Microbiota in Silkworms with Different Dietary Patterns

**Figure 1:**
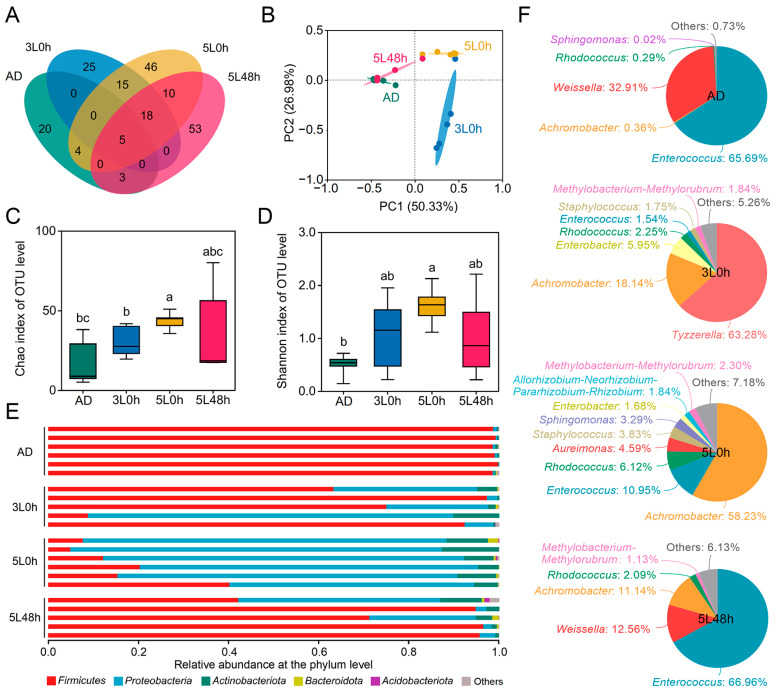
The gut microbiota structure of silkworms with different dietary patterns. Each gut sample came from five female and five male larvae at hour 72 of the fifth instar. AD, all instar silkworms were fed artificial diets (*n* = 6). 3L 0 h, first to second instar silkworms were fed artificial diets, and third to fifth instar silkworms were fed fresh mulberry leaves (*n* = 5). 5L 0 h, first to fourth instar silkworms were fed artificial diets and fifth instar silkworms were fed fresh mulberry leaves (*n* = 6). 5L 48 h, silkworms before hour 48 of the fifth instar were fed artificial diets, and silkworms after hour 48 of the fifth instar were fed fresh mulberry leaves (*n* = 5). (**A**) Venn diagram of the four groups at the OTU level. (**B**) Principal coordinate analysis (PCoA) diagram. (**C**) Chao index of species richness and (**D**) Shannon index of community diversity. Significant differences between groups are indicated with different letters (Student’s *t*-test, *p* < 0.05). (**E**) Relative abundance of gut microbiota at the phylum level in different samples. (**F**) Pie chart showing the genus-level relative abundance of the gut microbiota for each sample group.

To investigate the effects of transitioning from artificial diets (ADs) to mulberry leaves (MLs) on the gut microbiota of silkworms, we compared the composition and abundance of gut microbiota in 72 h fifth instar larvae under three dietary transition patterns: first to second instars fed ADs and third to fifth instars fed MLs (3L 0 h group); first to fourth instars fed ADs and fifth instar fed MLs (5L 0 h group); and silkworms fed ADs before 48 h of the fifth instar and MLs after 48 h of the fifth instar (5L 48 h group). Silkworms fed ADs throughout all instars (AD group) were used as the control. The Venn diagram indicated that in the intestines of silkworms in the AD group, 3L 0 h group, 5L 0 h group, and 5L 48 h group, 32, 63, 96, and 89 OTUs were detected, respectively, among which 20, 25, 46, and 53 OTUs were unique to each group, respectively, and only five OTUs were common to all four groups (Figure 1A). These findings indicated differences in the gut microbiota composition of silkworms under different dietary patterns. PCoA at the OTU level revealed that the microbial structures of the 5L 48 h group and the AD group were highly similar, whereas the microbial structure of the 3L 0 h group showed significant differences with respect to the other three groups (Figure 1B). Comparative analysis of the diversity of the gut microbiota among groups indicated that compared with the AD group, the other three groups exhibited greater richness and diversity of the gut microbiota, and the 5L 0 h group showed the most significant differences (Figure 1C,D). These results suggested that the dietary transition from ADs to MLs increases the quantity and diversity of gut microbiota in silkworms and is influenced by the transition time to MLs.

The differences in the gut microbiota composition in silkworms were next investigated under different dietary patterns. Phylum level analysis indicated that the top three dominant phyla in all four groups were *Firmicutes*, *Proteobacteria*, and *Actinobacteria*, but the proportions slightly differed among groups (Figure 1E). The highest abundance of *Firmicutes* was observed in the AD, 3L 0 h, and 5L 48 h groups at 98.93%, 67.32%, and 80.02%, respectively, whereas the highest abundance of *Proteobacteria* (74.57%) was observed in the 5L 0 h group. Furthermore, the abundance of *Proteobacteria* in the 3L 0 h and 5L 48 h groups reached 28.77% and 15.20%, respectively, but was only 0.48% in the AD group. The differences between the 5L 0 h group and the 5L 48 h group indicated that the effects of dietary transition at different times on the intestinal microbiota in silkworm larvae significantly differed, even within the same developmental instar.

Further genus level analysis (Figure 1F) indicated that the gut microbiota structure of the AD group was highly similar to that of the 5L 48 h group and was composed primarily of *Enterococcus*, *Weissella*, and *Achromobacter*. *Enterococcus* was the dominant genus, with abundance of 65.69% and 66.96% in the AD and 5L 48 h groups, respectively. *Weissella* was the second most dominant genus, with abundance of 32.91% and 12.56% in the AD and 5L 48 h groups, respectively. The dominant genus in the 3L 0 h group was *Tyzzerella* (63.28%), the dominant genus in the 5L 0 h group was *Achromobacter* (58.23%), while the abundance values of *Enterococcus* in the 3L 0 h group and the 5L 0 h group were 1.54% and 10.95%, respectively. These findings indicated that the dietary transition from ADs to MLs significantly increased the diversity of the gut microbiota in silkworms and decreased the proportion of the opportunistic pathogen *Enterococcus*. 

**Figure 2:**
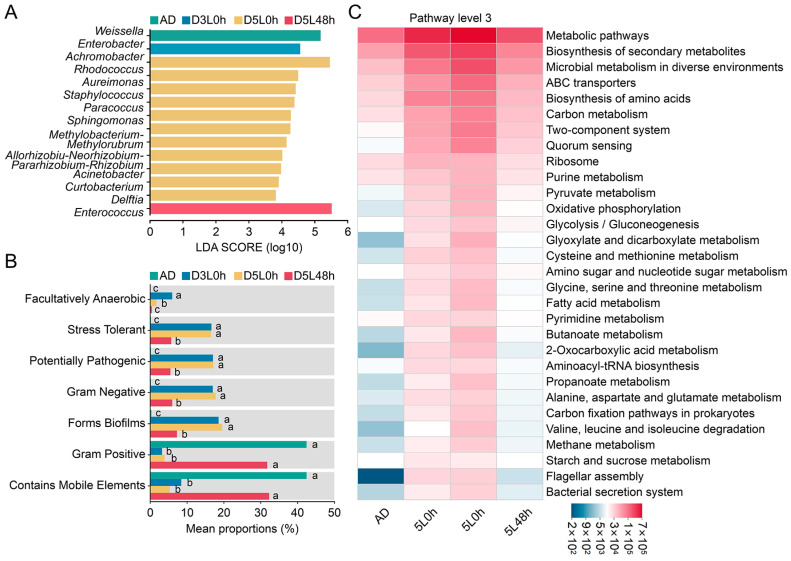
Differential analysis of the gut microbiota in silkworms under different dietary patterns. (**A**) LEfSe (LDA effect size) was used to analyze species differences at the genus level among groups. The LDA threshold was 2. The bar chart shows species with significant differences in abundance across multiple groups (multigroup comparison strategies: all-against-all). The greater the LDA score, the greater the influence of species abundance on the difference effect. (**B**) Microbial phenotype prediction of each group sample with BugBase. The bar chart shows the relative abundance of microbial phenotypes in each group (Kruskal–Wallis H test). A *p*-value < 0.05 indicated significant differences between groups, as shown with different letters. (**C**) Prediction of microbial functions for each group’s samples performed with PICRUSt. The color gradient of color blocks shows the variation in functional abundance among samples.

The microbial community abundance in silkworms showed significant differences in the proportions of *Firmicutes* and *Proteobacteria* between the 5L 0 h group and the other three groups at the phylum level, and a significant increase was observed in the proportion of *Actinobacteria* compared with the AD group (Figure S1A). At the genus level, significant differences were observed in the relative abundance of major bacterial genera in the three dietary transition groups compared with the AD group (Figure S1B). Linear discriminant analysis effect size (LEfSe) indicated 14 significantly differentially present genera among groups: one genus in the AD group, 3L 0 h group, and 5L 48 h group and 11 genera in the 5L 0 h group (Figure 2A). Our findings thus suggested that the dietary transition time substantially influences the gut microbiota of silkworms and may be associated with functional changes in the gut microbiota during different developmental stages.

We further investigated the potential effects of the gut microbiota in silkworms under different dietary patterns on host physiology. Using BugBase for phenotypic prediction of the gut microbiota (Figure 2B), we determined that the relative abundance of Gram-positive and mobile elements in the AD and 5L 48 h groups were significantly higher than that in the 3L 0 h and 5L 0 h groups, whereas the relative abundance of Gram-negative, potentially pathogenic, stress-tolerant, and biofilm-forming species was significantly lower in the AD and 5L 48 h groups than the 3L 0 h and 5L 0 h groups. The relative abundance of facultative anaerobes was significantly higher in the 3L 0 h group than the other groups, thus suggesting that differences in the gut microbiota of silkworms under different diet patterns might affect host resistance. On the basis of gut microbiota functional prediction analysis with PICRUSt2 (KEGG pathway level 3) (Figure 2C), we determined significant enrichment in metabolic pathways in the 3L 0 h, 5L 0 h, and 5L 48 h groups with respect to the AD group, most of which were associated with amino acid metabolism. Examples included “metabolic pathways”, “biosynthesis and metabolism of amino acids”, “carbon metabolism”, “ABC transporters”, “purine metabolism”, “pyruvate metabolism”, “glycine, serine, and threonine metabolism”, and “alanine, aspartate, and glutamate metabolism”, among which the 5L 0 h group had the highest relative abundance and was followed by the 3L 0 h group. These findings suggested that the differences in the gut microbiota after dietary transition might affect protein metabolism and silk protein synthesis in fifth instar silkworm larvae.

### 2.2. Dietary Patterns Affect the Development and Resistance of Silkworm Larvae

We next assessed the effects of these changes on larval development on the basis of the alterations in silkworm gut microbiota mediated by different dietary patterns. To eliminate the influence of developmental differences at different instar stages, we examined three additional groups: a 3L 24 h group (with ADs fed before 24 h of the third instar and mulberry leaves fed after 24 h of the third instar), 5L 24 h group (with ADs fed before 24 h of the fifth instar and mulberry leaves fed after 24 h of the fifth instar), and ML group (with all instars fed mulberry leaves). We sought to investigate the effects of dietary transition at different times during the third or fifth instar on the growth and development of silkworm larvae.

**Figure 3:**
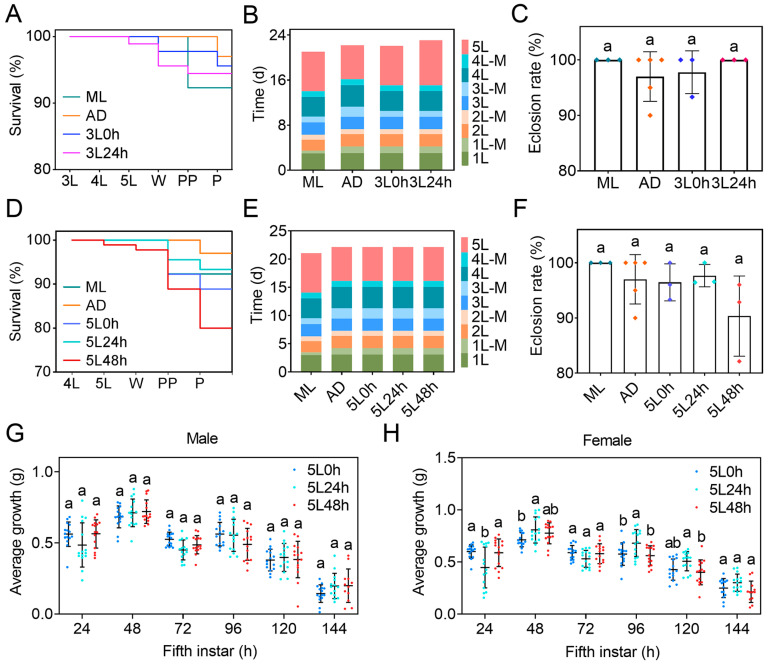
Effects of different dietary patterns on the growth and development of silkworms. (**A**–**C**) Effects of dietary transition starting from the third instar on the development of silkworms. (**A**) Survival rate. (**B**) Duration of all larval instars. (**C**) Eclosion rate (one-way ANOVA. *F* = 0.799; df = 3, 10; *p* = 0.522). (**D**–**F**) Effects of dietary transition starting from the fifth instar on the development of silkworms. (**D**) Survival rate. (**E**) Duration of all larval instars. (**F**) Eclosion rate (one-way ANOVA. *F* = 2.161; df = 4, 12; *p* = 0.136). (**G**,**H**) The 24-hour weight gain in male and female larvae. (**G**), Male (*F* = 2.045; df = 10, 248; *p* = 0.030). (**H**), Female (*F* = 4.870; df = 10, 248; *p* < 0.001). Two-way ANOVA. A *p*-value < 0.05 indicated significant differences between groups, as shown with different letters. Fifteen silkworms (of the same sex) with similar weight (±10%) and normal morphology were selected from the newly molted fifth instar larvae. The weight changes for each silkworm were tracked and investigated every 24 h. AD group, all instars fed artificial diets (ADs); ML group, all instars fed mulberry leaves (MLs); 3L 0 h group, first to second instars fed Ads and third to fifth instars fed MLs; 3L 24 h group, silkworms fed ADs before hour 24 of the third instar and MLs after hour 24 of the third instar; 5L 0 h group, first to fourth instars fed ADs and fifth instar fed MLs; 5L 24 h group, silkworms fed ADs before hour 24 of the fifth instar and MLs after hour 24 of the fifth instar; 5L 48 h group, silkworms fed ADs before hour 48 of the fifth instar and MLs after hour 48 of the fifth instar. W, wandering stage. PP, prepupal stage. P, pupal stage. 1L, 2L, 3L, 4L, and 5L represent the first instar, second instar, third instar, fourth instar, and fifth instar, respectively. 1L-M, 2L-M, 3L-M, and 4L-M represent the first molt, second molt, third molt, and fourth molt, respectively.

A vitality survey indicated that the survival rate of the AD group until the pupal stage was 97.00%, whereas that in the ML group was 92.31%. The vitality of the silkworms in the 3L 0 h and 3L 24 h groups was intermediate between those in the AD group and the ML group, with rates of 95.56% and 94.44%, respectively (Figure 3A). The larval development of silkworms in the AD group was similar to that in the ML group, whereas the duration of the fifth instar in the 3L 0 h group and 3L 24 h group was 2 days and 3 days longer, respectively, than that in the AD group (Figure 3B). In addition, no significant difference was observed in the eclosion rate among groups (Figure 3C). Thus, the changes in dietary patterns starting from the third instar stage did not significantly affect the vitality of the silkworms, but the developmental duration during different larval stages clearly differed.

Further investigation was conducted to assess the effects of dietary transition at different times during the fifth instar on the growth and development of silkworms. The survival rates of the 5L 0 h group, 5L 24 h group, and 5L 48 h group were 88.89%, 93.33%, and 80.00%, respectively (Figure 3D). The duration of the fifth instar in these three dietary transition groups was approximately 1 day longer than that in the AD group (Figure 3E), whereas the eclosion rate showed no significant differences with respect to both the AD and ML groups (Figure 3F). In addition, no significant differences were observed in body weight and 24-hour weight gain among male larvae in the 5L 0 h group, 5L 24 h group, and 5L 48 h group (Figure 3G and Figure S2A). However, the weight among female larvae in the 5L 24 h group was significantly higher than that in the 5L 48 h group on day 4 of the fifth instar, and the 24-hour weight gain on day 4 and day 5 of the fifth instar was significantly higher than that in the 5L 0 h group and the 5L 48 h group (Figure 3H and Figure S2B). These results indicated that the effects of dietary transition at different times within the same instar on the growth and development of silkworms varied, and female larvae were more sensitive than males in their response.

**Figure 4:**
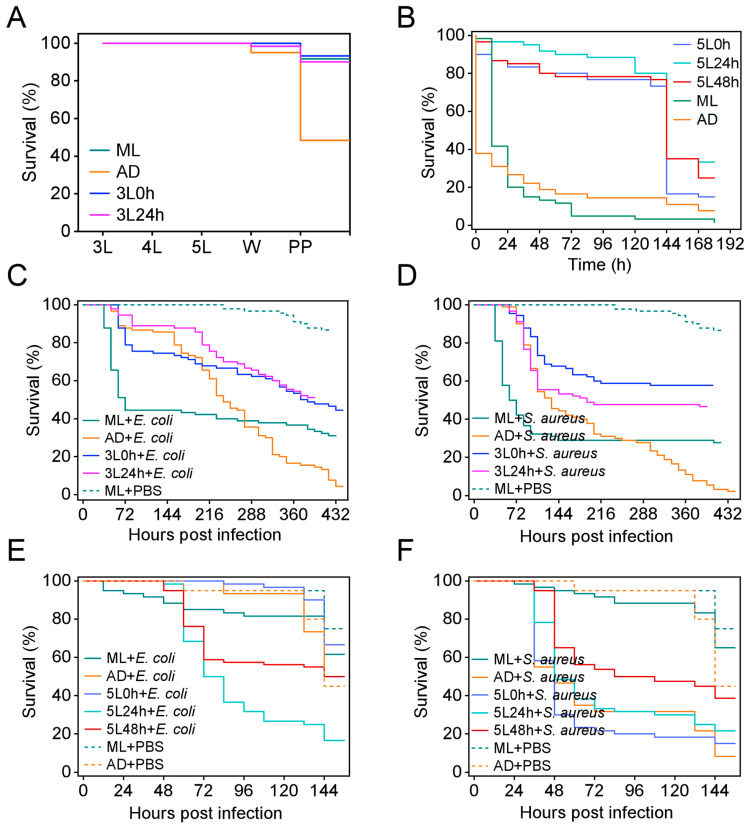
Effects of different dietary patterns on the resistance of silkworms. (**A**,**B**) Survival rates of silkworms with dietary transition from the third or fifth instar under high temperature stress. (**A**) Each sample consisted of 15 male and 15 female third instar silkworm larvae, with three replicates per group. Hour 40 third instar larvae were placed in a 40 °C incubator for 6 h (without food), then transferred back to the standard feeding conditions of 25 °C. (**B**) Each sample consisted of 10 male and 10 female fifth instar silkworm larvae, with three replicates per group. Hour 72 fifth instar larvae were placed in a 40 °C incubator for 12 h (without food), then transferred back to the standard feeding conditions of 25 °C. (**C**,**D**) Survival rates of the third instar larvae infected with *E. coli* or *S. aureus*. Two microliters of *E. coli* (OD_450_ = 1.6) or *S. aureus* (OD_450_ = 0.2) was injected into the dorsal vessel of hour 40 third instar larvae through a capillary, and the ML larvae were injected with 2 μL PBS as a control. Each sample consisted of 15 male and 15 female silkworm larvae, with three sample replicates per group. (**E**,**F**) Survival rates of the fifth instar larvae infected with *E. coli* or *S. aureus*. Ten microliters of *E. coli* (OD_450_ = 1.6) or *S. aureus* (OD_450_ = 0.2) was injected into the dorsal vessel of hour 72 fifth instar larvae through a capillary, and the AD and ML larvae were injected with 10 μL PBS as control. Each sample consisted of 10 male and 10 female silkworm larvae, with three sample replicates per group.

Previous studies have indicated that the changes in the gut microbiota in silkworms induced by dietary transition are associated with immunity and amino acid metabolism [30]. To determine the effects of this dietary pattern change on the heat resistance of silkworm larvae, we shocked 40 h third instar larvae with 40 °C hot air for 6 h. No significant changes in the viability of the larvae were observed in all groups during the entire larval stage, but the survival rate in the AD group significantly decreased after the wandering stage and was only 48.33%, whereas that in the 3L 0 h group and 3L 24 h group reached 93.33% and 90.00%, respectively—values similar to those in the ML group (91.67%) (Figure 4A). After the 72 h fifth instar larvae were shocked with 40 °C hot air for 12 h, the survival rate of the AD group sharply decreased to 26.67% after 24 h, whereas that of the 5L 0 h group, 5L 24 h group, and 5L 48 h group exceeded 70% before 132 h but significantly decreased after 144 h; the final survival rates were 15%, 33.33%, and 25%, respectively, all of which were higher than the 7.78% in the AD group (Figure 4B). These findings indicated that the transition from ADs to MLs in the third or fifth instar enhanced the resistance of silkworm larvae under high-temperature stress, and the effects of the dietary transition in 24 h fifth instar larvae were more favorable than those in 0 h and 48 h fifth instar larvae.

Further evaluation was conducted on the differences in the resistance of silkworms to bacterial infections under different dietary patterns. After injection of *E. coli* or *S. aureus* into the dorsal vein in 40 h third instar larvae, regardless of the pathogen, the survival rate was higher in the AD group than the ML group in the early stages of infection but reversed in the later stages. The survival rates of the 3L 0 h group and the 3L 24 h group after infection with *E. coli* and *S. aureus* were higher than those of the ML and AD groups, and the 3L 0 h group showed a higher survival rate than the 3L 24 h group after infection with *S. aureus* (Figure 4C,D). Furthermore, after injection of *E. coli* or *S. aureus* into 72 h fifth instar larvae, the survival rate of the 5L 48 h group infected with *E. coli* was similar to that of the AD group but lower than that of the 5L 0 h group (Figure 4E). In contrast, the survival rates of the 5L 0 h, 5L 24 h, and 5L 48 h groups infected with *S. aureus* were intermediate between those of the ML group and the AD group, and the 5L 48 h group showed the highest survival (Figure 4F). These results indicated that under the investigated dietary patterns of transition from ADs to MLs at different times during the third or fifth instar stages, silkworm larvae exhibited varying resistance to different bacteria but generally outperformed AD-reared silkworms.

### 2.3. Different Dietary Patterns Affect Cocoon Silk Yield in Silkworms

**Figure 5:**
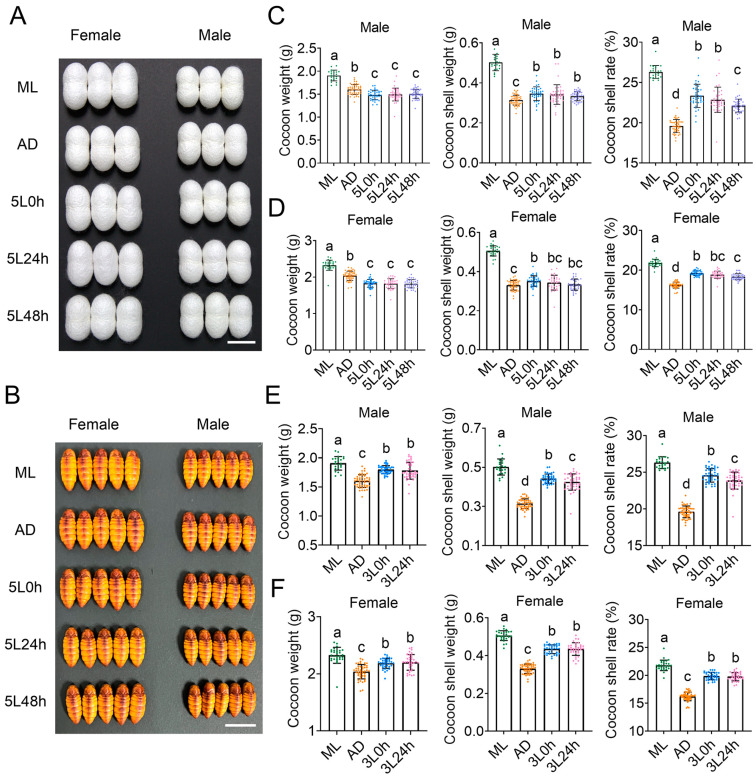
Effects of different dietary patterns on the silk yield of silkworms. (**A**,**B**) Images of silkworm cocoons and pupae. Scale bars: 2 cm. (**C**,**D**) Effects of dietary transition starting from the fifth instar on silk yield. (**C**) Male (*n* = 23, 48, 38, 40, 42). (**D**) Female (*n* = 29, 48, 40, 33, 37). Cocoon weight (*F* = 101.4; df = 4, 182; *p* < 0.001). Cocoon shell weight (*F* = 207.4; df = 4, 182; *p* < 0.001). Cocoon shell rate (*F* = 230.8; df = 4, 182; *p* < 0.001). (**E**,**F**) Effects of dietary transition starting from the third instar on silk yield. (**E**) Male (*n* = 23, 48, 43, 41). Cocoon weight (*F* = 46.25; df = 3, 151; *p* < 0.001). Cocoon shell weight (*F* = 217.1; df = 3, 151; *p* < 0.001). Cocoon shell rate (*F* = 354.1; df = 3, 151; *p* < 0.001). (**F**) Female (*n* = 29, 48, 37, 36). Cocoon weight (*F* = 34.57; df = 3, 146; *p* < 0.001). Cocoon shell weight (*F* = 279.2; df = 3, 146; *p* < 0.001). Cocoon shell rate (*F* = 361.4; df = 3, 146; *p* < 0.001). A *p*-value < 0.05 indicates significant differences between groups, as shown with different letters. One-way ANOVA.

Because the most important amino acid metabolic activity in fifth instar silkworms is silk protein synthesis, we evaluated the effects of dietary transition at the fifth instar on cocoon silk production. The cocoon size in the AD group was the smallest, but the size of the pupae was greater than observed in the three dietary transition groups (Figure 5A,B). The ML group had the highest cocoon weight (in both sexes) and was followed by the AD group. In addition, the cocoon shell weight of males in the 5L 0 h group, 5L 24 h group, and 5L 48 h group, as well as the females in the 5L 0 h group, were significantly greater than those in the AD group; however, no significant differences were observed among the three dietary transition groups of the same sex. Importantly, the cocoon shell rate, the most representative indicator of silk protein synthesis efficiency in silkworms, was significantly higher in the 5L 0 h, 5L 24 h, and 5L 48 h groups than the AD group (in both sexes). Among them, no significant difference was observed in the cocoon shell rate between the 5L 0 h and 5L 24 h groups, and both rates were superior to that in the 5L 48 h group (Figure 5C,D).

The dietary transition from ADs to MLs, starting from the third instar larvae, showed similar trends in effects on cocoon silk yield. In both sexes, the 3L 0 h group and 3L 24 h group exhibited greater cocoon weight, cocoon shell weight, and cocoon shell rate than the AD group, although these values were lower than those in the ML group. In addition, the cocoon shell weight and cocoon shell rate for male larvae were significantly higher in the 3L 0 h group than the 3L 24 h group (Figure 5E,F). These results indicated that even within the same instar, differences exist in the effects of different dietary transition times on the efficiency of silk protein synthesis in silkworms.

## 3. Discussion

### 3.1. Dietary Transition Reshapes the Gut Microbiota of Silkworms

Habitual dietary changes affect the long-term health of animals through gut microbiota-dependent mechanisms [31,32]. For instance, metabolic dysfunction induced by an HFD is associated with alterations in gut microbial composition and metabolic products [19,33]. Berberine has been shown to effectively alleviate gut dysbiosis in mice fed an HFD; to increase the abundance of beneficial microorganisms such as *Akkermansia* and *Parabacteroides*; and to directly inhibit HFD-associated colorectal cancer through gut microbiota-regulated lyso-phosphatidylcholine [34]. A spirulina platensis polysaccharides intervention has been found to alter the relative abundance of intestinal microorganisms and decrease cecal levels of propionic acid in HFD-fed rats, thereby attenuating lipid and carbohydrate metabolism disorders induced by the HFD [22,35].

For silkworms, MLs are considered the most suitable diet, whereas AD is a nutritionally imbalanced diet [16,36]. Studies have shown that the dietary pattern of MLs shapes the formation of similar and stable gut microbiota among individual silkworms and is crucial for their health [28]. Our previous research has indicated that silkworms fed ADs exhibit not only diminished gut microbiota diversity but also significant changes in the abundance of dominant species [30]. Recent studies have suggested that enrichment in *Lactobacillus*, *Weissella*, and *Enterococcus* under AD feeding conditions is associated with poor health in silkworms [15]. In this study, the diversity and abundance of gut microbiota were greater in silkworms fed MLs instead of ADs, regardless of the duration of the substitution. The relative abundance of *Weissella* and opportunistic pathogenic bacteria *Enterococcus* significantly decreased with increasing duration of mulberry leaf feeding, whereas the trace levels of *Achromobacter* and *Rhodococcus* in the AD group were markedly higher in the dietary transition group. Notably, the dominant bacteria in the 5L 0 h and 5L 24 h groups were *Achromobacter* and *Enterococcus*, respectively. The structure of the gut microbiota in the 5L 24 h group, which had a shorter duration of mulberry leaf intake, was close to that of the AD group, whereas the diversity of the gut microbiota was higher in the 5L 0 h group. Furthermore, the dominant genus in the 3L 0 h group, which had the longest duration of mulberry leaf intake, was *Tyzzerella*, with gut microbiota diversity and abundance intermediate between those of the 5L 0 h and 5L 24 h groups. Therefore, the duration of dietary switching significantly affects the reshaping of silkworm gut microbiota.

Alterations in gut microbial composition and dominant bacterial abundance play crucial roles in intestinal health [37,38,39]. In this study, the midgut tissues of silkworm larvae in both the AD group and the three dietary transition groups exhibited an intact morphological structure, with evenly distributed, neatly arranged goblet cells and cylindrical cells. However, the vacuoles in goblet cells were noticeably larger in the 3L 0 h and 5L 0 h groups than in the AD group and the 5L 48 h group (Figure S3A). Moreover, the lipase activity in the intestinal fluid in the 3L 0 h and 5L 0 h groups was higher than that in the AD group (Figure S3B). We therefore speculate that dietary transition from ADs to MLs improves the activity of silkworm intestinal cells, thereby facilitating nutrient digestion and absorption [40].

### 3.2. Dietary Transition Increases Resistance and Silk Production Efficiency in Silkworms

The gut microbiota and microbial metabolites significantly affect various physiological functions of hosts, including insects [41,42]. In this study, significant changes in the function of the gut microbiota were observed in the dietary transition groups compared with the AD group and were associated with the duration of dietary conversion. The gut microbiota in the dietary transition groups were associated with more beneficial nutrient metabolism and transport than observed in the AD group. For example, increased abundance of *Achromobacter* helps the host degrade cellulose [43,44], and increased abundance of *Rhodococcus* contributes to the metabolism of sugars and aromatic compounds [45,46]. These changes in the gut microbiota synergistically promoted protein synthesis, thus resulting in significantly greater silk production efficiency in the dietary transition groups than the AD group. In addition, the higher functional abundance of amino acid metabolism pathways in the 5L 0 h group of silkworms than the 5L 48 h group resulted in superior silk production efficiency. The degree of improvement in silk protein synthesis efficiency varied among diet conversion times, even within the same instar, a finding potentially attributable to the ability of the silkworm gut microbiota structure to rapidly adapt to changes in diet.

Previous research has shown that diet directly or indirectly influences the host’s immune response [47,48,49]. In this study, the transition from ADs to MLs enhanced the high-temperature resistance and antibacterial ability of silkworm larvae. The duration of the dietary substitution influenced the extent of this improvement. These findings are consistent with the increased abundance of immune-associated pathways predicted by BugBase and the significant increase in the abundance of *Rhodococcus*, which plays a role in regulating insect immune defense [50]. The gut microbiota also mediates the regulation of host health by converting dietary compounds into various bioactive metabolites [51,52,53]. Dietary fiber can be metabolized by the gut microbiota into short-chain fatty acids, such as acetate, propionate, and butyrate, which participate in regulating the host’s immune response and metabolic health [54,55,56]. In this study, the abundance of fatty acid synthesis, propionic acid metabolism, and butyric acid metabolism functions was enhanced in the dietary transition groups. We speculated that the structure and function of the silkworm gut microbiota changed with the dietary transition from ADs to MLs, thus improving the high-temperature resistance and antibacterial ability of silkworms.

Previous studies have demonstrated that the efficient increase in body weight and silk production of silkworms following the transition from artificial diet to mulberry leaf rearing is associated with overall metabolic changes [57]. The findings of this study suggest that changes in gut microbiota may also play an important role, which holds positive implications for enhancing the rearing effect of artificial diets from a probiotic perspective in the future. In summary, using the silkworm model, this study has initially discovered the significant impact of dietary transitions on gut microbiota, particularly the variations in the extent of gut microbiota remodeling depending on the duration of the dietary transition. Future research will explore how the reshaping of gut microbiota induced by dietary transitions affects the health of the host.

## 4. Materials and Methods

### 4.1. Animal Preparation

The silkworm strain (*Jingsong* × *Haoyue*) used in this study was provided by Soochow University. The silkworm larvae were reared at a temperature of 25 °C to 28 °C, a humidity of 60% to 85%, and a 12-hour/12-hour light/dark cycle, and were fed fresh mulberry leaves or an AD. The AD used in this study was developed by our research laboratory [30] rather than being a commercially purchased silkworm artificial diet. Sterile wet diets were obtained by steaming at high temperature (100 °C, 1 h). Silkworms of all instars fed ADs comprised the AD group, and silkworms of all instars fed MLs comprised the ML group. The dietary transition groups are denoted as follows, according to the start time of the transition from ADs to MLs: 3L 0 h group, 3L 24 h group, 5L 0 h group, 5L 24 h group, and 5L 48 h group.

### 4.2. Investigation of Growth and Development

To investigate the developmental duration, survival rate, and eclosion rate of silkworm larvae under standard rearing conditions, we randomly selected 90 larvae from different dietary pattern groups for tracking and investigation. The first instar, second instar, third instar, fourth instar, and fifth instar are denoted 1L, 2L, 3L, 4L, and 5L, respectively, and the first molt, second molt, third molt, and fourth molt are denoted 1L-M, 2L-M, 3L-M, and 4L-M, respectively. W, PP, and P represent the wandering stage, prepupal stage, and pupal stage, respectively. In investigations of the 24-hour weight gain of fifth instar silkworms, each sample represents the average of one male and one female silkworm, with 15 replicates per group. Investigations of the silk yield of silkworms were conducted by sex, with 20–50 replicates per group.

### 4.3. Investigation of Silkworm Resistance to High Temperatures

Study of the survival of third instar silkworms under high-temperature stress: When the silkworms developed to hour 40 of the third instar, larvae from the AD group, ML group, and dietary transition groups (3L 0 h group and 3L 24 h group) were transferred to a constant temperature incubator set at 40 °C. After 6 h, the larvae were transferred back to the standard feeding conditions of 25 °C, and the number of dead silkworms was recorded every 12 h (with three independent replicates per group, with each replicate consisting of fifteen male and fifteen female silkworms).

Study of the survival of fifth instar silkworms under high-temperature stress: When the silkworms developed to hour 72 of the fifth instar, larvae from the AD group, ML group, and dietary transition groups (5L 0 h group, 5L 24 h group, and 5L 48 h group) were transferred to a constant temperature incubator set at 40 °C. After 12 h, the larvae were returned to a normal rearing environment, and the number of dead silkworms was recorded every 12 h (with three independent replicates per group, with each replicate consisting of ten male and ten female silkworms).

### 4.4. Investigation of the Antibacterial Ability of Silkworms

Preparation of pathogenic bacteria: According to previously described methods [58], *Staphylococcus aureus* (*S. aureus*) and *Escherichia coli* (*E. coli*) were separately cultured in LB medium for 10–12 h (37 °C, 180 r/min) until reaching logarithmic growth phase. After centrifugation (5000 r/min, 2 min), the bacteria were collected and subsequently washed twice with phosphate-buffered saline (PBS). The bacterial suspension was diluted with PBS, and the concentrations of *S. aureus* (OD_450_ = 0.2) and *E. coli* (OD_450_ = 1.6) were determined with a microplate spectrophotometer (Eon, BioTek, Winooski, VT, USA).

Investigation of the survival rate of third instar larvae after bacterial infection: Hour 40 third instar larvae from the AD group, ML group, and dietary transition groups (3L 0 h group and 3L 24 h group) were selected. Subsequently, 2 μL of *S. aureus* or *E. coli* was injected into the larval dorsal vessel with a capillary tube. The ML larvae were injected with 2 μL PBS as a control. Three independent replicates per group were assessed, each consisting of fifteen male and fifteen female silkworms.

Investigation of the survival rate of fifth instar larvae after bacterial infection: Hour 72 fifth instar larvae from the AD group, ML group, and dietary transition groups (5L 0 h group, 5L 24 h group, and 5L 48 h group) were selected. Subsequently, 10 μL of *S. aureus* or *E. coli* was injected into the larval dorsal vessel with a capillary tube. The AD and ML larvae were injected with 10 μL PBS as a control. Three independent replicates per group were assessed, each consisting of ten male and ten female silkworms.

After inoculation with bacteria, the silkworms were returned to normal rearing conditions, and the number of surviving larvae was counted every 12 h.

### 4.5. Hematoxylin and Eosin Staining

Male 72 h fifth instar silkworm larvae (AD group, 3L 0 h group, 5L 0 h group, and 5L 48 h group) were selected. The midgut was dissected and washed with 0.7% physiological saline, then fixed with 4% paraformaldehyde. The fixed midgut was dehydrated in a 50–100% ethanol gradient and rendered transparent with xylene. Subsequently, the midgut tissue was embedded in melted paraffin, solidified, and sliced into 10 μm thick sections. The midgut sections were then deparaffinized with xylene, dehydrated in a 100–50% ethanol gradient, and stained with hematoxylin and eosin. Finally, the stained sections were dehydrated in a 95–100% ethanol gradient, observed, and photographed under a microscope (Olympus BX51, Olympus Corporation, Tokyo, Japan).

### 4.6. Detection of Enzymatic Activity in Intestinal Fluid

Male and female 72 h fifth instar silkworm larvae (AD group, 3L 0 h group, 5L 0 h group, 5L 48 h group) were selected (satiated state). The entire digestive tract was dissected and removed, then thoroughly rinsed with 0.7% saline solution. After the removal of surface moisture, all intestinal fluids were collected. The intestinal fluid from five male larvae and five female larvae was mixed and served as one testing sample, and the analysis was repeated for three samples per group. Subsequently, the activity of α-amylase and lipase in each sample was measured with reagent kits (C016-1-1; A054-2-1, Nanjing Jiancheng, Nanjing, China) according to the manufacturer’s instructions. The absorbance of each sample was measured with a microplate spectrophotometer (Eon, BioTek, Winooski, VT, USA).

### 4.7. Sequencing and Analysis of Gut Microbiota

Silkworm larvae at hour 72 of the fifth instar (AD group, 3L 0 h group, 5L 0 h group, 5L 48 h group) were selected. The surfaces of the larvae were disinfected with 75% ethanol, and the entire digestive tract (including its contents) was dissected and obtained in a sterile environment. The digestive tracts from five male larvae and five female larvae were mixed and served as one detection sample; the analysis was repeated for six samples per group.

Genomic DNA extraction, Illumina Novaseq 6000 platform sequencing, and library construction were performed by Majorbio (Shanghai, China). Data analysis was performed on the Majorbio cloud platform (www.majorbio.com, accessed on 28 October 2022). Operational taxonomic unit (OTU) clustering analysis of sequences at a 97% similarity level was performed with Uparse (version 7.0.1090, http://drive5.com/uparse/, accessed on 28 October 2022). A Venn diagram was used to illustrate the number of shared and unique OTUs among groups. R language (Version 3.3.1) was used for principal coordinate analysis (PCoA) and visualization of the similarity or dissimilarity of community composition between samples. Mothur (version v.1.30.2, https://mothur.org/wiki/calculators/, accessed on 28 October 2022) was used to calculate the alpha diversity indices, including the Chao index and Ace index, which represent species richness and community diversity at the OTU level, respectively. With the RDP classifier (version 2.13, http://sourceforge.net/projects/rdp-classifier/, accessed on 6 February 2023) and the Silva 16S rRNA gene database used for alignment, we obtained species classification annotations with a threshold of 70%. The composition of the community at the phylum and genus levels was analyzed for each sample. Linear discriminant analysis effect size (LEfSe) software (http://huttenhower.sph.harvard.edu/galaxy/root?tool_id=lefse_upload, accessed on 2 September 2023) was used to detect significant differences in microbial species between groups. BugBase software (https://bugbase.cs.umn.edu/index.html, accessed on 6 February 2023) was used to predict microbial traits, and PICRUSt2 software (version 2.2.0, http://huttenhower.sph.harvard.edu/galaxy, accessed on 5 December 2022) was used to predict the Kyoto Encyclopedia of Genes and Genomes (KEGG) metabolic pathways of the microbiota.

### 4.8. Data Analysis

Survival curves were plotted with OriginPro 2021 (V9.8.5.204, OriginLab, Northampton, MA, USA), and statistical analysis and graphing of data, such as developmental time, eclosion rate, body weight, cocoon yield, and digestive enzyme activity, were performed with GraphPad Prism 8 software (V8.0.2.263, GraphPad, San Diego, CA, USA). Significance testing was conducted with an analysis of variance, with a statistical significance threshold set at *p* < 0.05. Additionally, all percentage data were transformed with the arcsine square root transformation before statistical significance analysis.

## 5. Conclusions

The transition of the habitual diet of silkworms from artificial diets to fresh mulberry leaves increased the diversity and abundance of the silkworm gut microbiota. The relative abundance of *Weissella* and *Enterococcus* decreased, whereas that of *Achromobacter* and *Rhodococcus* increased. The transition from artificial diets to fresh mulberry leaves improved the silkworms’ immune resistance and silk production efficiency and was associated with the period of dietary transition. Thus, a preliminary silkworm model for studying the relationship between habitual dietary changes and the gut microbiota has been established.

## Data Availability

The data presented in this study are available upon request from the corresponding author.

## Supplementary Materials

The following supporting information can be downloaded at: https://www.mdpi.com/article/10.3390/ijms25031722/s1.

## References

[1] Wolter M., Grant E.T., Boudaud M., Steimle A., Pereira G.V., Martens E.C., Desai M.S. (2021). Leveraging diet to engineer the gut microbiome. Nat. Rev. Gastroenterol. Hepatol..

[2] Zmora N., Suez J., Elinav E. (2019). You are what you eat: Diet, health and the gut microbiota. Nat. Rev. Gastroenterol. Hepatol..

[3] Azzoni R., Marsland B.J. (2022). The lung-brain axis: A new frontier in host-microbe interactions. Immunity.

[4] Fan Y., Pedersen O. (2021). Gut microbiota in human metabolic health and disease. Nat. Rev. Microbiol..

[5] Yuan S., Sun Y., Chang W., Zhang J., Sang J., Zhao J., Song M., Qiao Y., Zhang C., Zhu M. (2023). The silkworm (*Bombyx mori*) gut microbiota is involved in metabolic detoxification by glucosylation of plant toxins. Commun. Biol..

[6] Chen B., Zhang N., Xie S., Zhang X., He J., Muhammad A., Sun C., Lu X., Shao Y. (2020). Gut bacteria of the silkworm *Bombyx mori* facilitate host resistance against the toxic effects of organophosphate insecticides. Environ. Int..

[7] von Frieling J., Faisal M.N., Sporn F., Pfefferkorn R., Nolte S.S., Sommer F., Rosenstiel P., Roeder T. (2020). A high-fat diet induces a microbiota-dependent increase in stem cell activity in the *Drosophila* intestine. PLoS Genet..

[8] Yao S., Zhao Y., Chen H., Sun R., Chen L., Huang J., Yu Z., Chen S. (2023). Exploring the Plasticity of Diet on Gut Microbiota and Its Correlation with Gut Health. Nutrients.

[9] Spencer C.N., McQuade J.L., Gopalakrishnan V., McCulloch J.A., Vetizou M., Cogdill A.P., Khan M.A.W., Zhang X., White M.G., Peterson C.B. (2021). Dietary fiber and probiotics influence the gut microbiome and melanoma immunotherapy response. Science.

[10] Perler B.K., Friedman E.S., Wu G.D. (2023). The Role of the Gut Microbiota in the Relationship Between Diet and Human Health. Annu. Rev. Physiol..

[11] de Vos W.M., Tilg H., Van Hul M., Cani P.D. (2022). Gut microbiome and health: Mechanistic insights. Gut.

[12] Kolodziejczyk A.A., Zheng D., Elinav E. (2019). Diet-microbiota interactions and personalized nutrition. Nat. Rev. Microbiol..

[13] Tong X., Han M.J., Lu K., Tai S., Liang S., Liu Y., Hu H., Shen J., Long A., Zhan C. (2022). High-resolution silkworm pan-genome provides genetic insights into artificial selection and ecological adaptation. Nat. Commun..

[14] Wang D., Dong Z., Zhang Y., Guo K., Guo P., Zhao P., Xia Q. (2017). Proteomics Provides Insight into the Interaction between Mulberry and Silkworm. J. Proteome Res..

[15] Qin L., Qi J., Shen G., Qin D., Wu J., Song Y., Cao Y., Zhao P., Xia Q. (2022). Effects of Microbial Transfer during Food-Gut-Feces Circulation on the Health of *Bombyx mori*. Microbiol. Spectr..

[16] Li J., Deng J., Deng X., Liu L., Zha X. (2023). Metabonomic Analysis of Silkworm Midgut Reveals Differences between the Physiological Effects of an Artificial and Mulberry Leaf Diet. Insects.

[17] Yin X., Zhang Y., Yu D., Li G., Wang X., Wei Y., He C., Liu Y., Li Y., Xu K. (2023). Effects of artificial diet rearing during all instars on silk secretion and gene transcription in *Bombyx mori* (Lepidoptera: Bombycidae). J. Econ. Entomol..

[18] Yang M., Qi X., Li N., Kaifi J.T., Chen S., Wheeler A.A., Kimchi E.T., Ericsson A.C., Rector R.S., Staveley-O’Carroll K.F. (2023). Western diet contributes to the pathogenesis of non-alcoholic steatohepatitis in male mice via remodeling gut microbiota and increasing production of 2-oleoylglycerol. Nat. Commun..

[19] Frazier K., Kambal A., Zale E.A., Pierre J.F., Hubert N., Miyoshi S., Miyoshi J., Ringus D.L., Harris D., Yang K. (2022). High-fat diet disrupts REG3γ and gut microbial rhythms promoting metabolic dysfunction. Cell Host Microbe.

[20] Dapa T., Ramiro R.S., Pedro M.F., Gordo I., Xavier K.B. (2022). Diet leaves a genetic signature in a keystone member of the gut microbiota. Cell Host Microbe.

[21] Suriano F., Nyström E.E.L., Sergi D., Gustafsson J.K. (2022). Diet, microbiota, and the mucus layer: The guardians of our health. Front. Immunol..

[22] Li T.T., Huang Z.R., Jia R.B., Lv X.C., Zhao C., Liu B. (2021). Spirulina platensis polysaccharides attenuate lipid and carbohydrate metabolism disorder in high-sucrose and high-fat diet-fed rats in association with intestinal microbiota. Food Res. Int..

[23] Wastyk H.C., Fragiadakis G.K., Perelman D., Dahan D., Merrill B.D., Yu F.B., Topf M., Gonzalez C.G., Van Treuren W., Han S. (2021). Gut-microbiota-targeted diets modulate human immune status. Cell.

[24] Lai H., Li Y., He Y., Chen F., Mi B., Li J., Xie J., Ma G., Yang J., Xu K. (2023). Effects of dietary fibers or probiotics on functional constipation symptoms and roles of gut microbiota: A double-blinded randomized placebo trial. Gut Microbes.

[25] Hyoju S.K., Adriaansens C., Wienholts K., Sharma A., Keskey R., Arnold W., van Dalen D., Gottel N., Hyman N., Zaborin A. (2020). Low-fat/high-fibre diet prehabilitation improves anastomotic healing via the microbiome: An experimental model. Br. J. Surg..

[26] Gill S.K., Rossi M., Bajka B., Whelan K. (2021). Dietary fibre in gastrointestinal health and disease. Nat. Rev. Gastroenterol. Hepatol..

[27] Wang G., Jiang G., Peng R., Wang Y., Li J., Sima Y., Xu S. (2023). Multi-omics integrative analysis revealed characteristic changes in blood cell immunity and amino acid metabolism in a silkworm model of hyperproteinemia. Int. J. Biol. Macromol..

[28] Chen B., Du K., Sun C., Vimalanathan A., Liang X., Li Y., Wang B., Lu X., Li L., Shao Y. (2018). Gut bacterial and fungal communities of the domesticated silkworm (*Bombyx mori*) and wild mulberry-feeding relatives. ISME J..

[29] Shao Y., Mason C.J., Felton G.W. (2023). Toward an Integrated Understanding of the Lepidoptera Microbiome. Annu. Rev. Entomol..

[30] Dong H.L., Zhang S.X., Chen Z.H., Tao H., Li X., Qiu J.F., Cui W.Z., Sima Y.H., Cui W.Z., Xu S.Q. (2018). Differences in gut microbiota between silkworms (*Bombyx mori*) reared on fresh mulberry (*Morus alba* var. multicaulis) leaves or an artificial diet. RSC Adv..

[31] Wang H., Gou W., Su C., Du W., Zhang J., Miao Z., Xiao C., Jiang Z., Wang Z., Fu Y. (2022). Association of gut microbiota with glycaemic traits and incident type 2 diabetes, and modulation by habitual diet: A population-based longitudinal cohort study in Chinese adults. Diabetologia.

[32] De Angelis M., Garruti G., Minervini F., Bonfrate L., Portincasa P., Gobbetti M. (2019). The Food-gut Human Axis: The Effects of Diet on Gut Microbiota and Metabolome. Curr. Med. Chem..

[33] Yang J., Wei H., Zhou Y., Szeto C.H., Li C., Lin Y., Coker O.O., Lau H.C.H., Chan A.W.H., Sung J.J.Y. (2022). High-Fat Diet Promotes Colorectal Tumorigenesis Through Modulating Gut Microbiota and Metabolites. Gastroenterology.

[34] Chen H., Ye C., Wu C., Zhang J., Xu L., Wang X., Xu C., Zhang J., Guo Y., Yao Q. (2023). Berberine inhibits high fat diet-associated colorectal cancer through modulation of the gut microbiota-mediated lysophosphatidylcholine. Int. J. Biol. Sci..

[35] Jiang G.H., Wang G., Luo C., Wang Y.F., Qiu J.F., Peng R.J., Sima Y.H., Xu S.Q. (2023). Mechanism of hyperproteinemia-induced damage to female reproduction in a genetic silkworm model. iScience.

[36] Zhang Z.J., Zhang S.S., Niu B.L., Ji D.F., Liu X.J., Li M.W., Bai H., Palli S.R., Wang C.Z., Tan A.J. (2019). A determining factor for insect feeding preference in the silkworm, *Bombyx mori*. PLoS Biol..

[37] Lee J.Y., Tsolis R.M., Bäumler A.J. (2022). The microbiome and gut homeostasis. Science.

[38] Fassarella M., Blaak E.E., Penders J., Nauta A., Smidt H., Zoetendal E.G. (2021). Gut microbiome stability and resilience: Elucidating the response to perturbations in order to modulate gut health. Gut.

[39] Abou-Samra E., Hickey Z., Aguilar O.A., Scur M., Mahmoud A.B., Pyatibrat S., Tu M.M., Francispillai J., Mortha A., Carlyle J.R. (2019). NKR-P1B expression in gut-associated innate lymphoid cells is required for the control of gastrointestinal tract infections. Cell Mol. Immunol..

[40] Xu K., Lan H., He C., Wei Y., Lu Q., Cai K., Yu D., Yin X., Li Y., Lv J. (2022). Toxicological effects of trace amounts of pyriproxyfen on the midgut of non-target insect silkworm. Pestic. Biochem. Physiol..

[41] Grenier T., Leulier F. (2020). How commensal microbes shape the physiology of Drosophila melanogaster. Curr. Opin. Insect Sci..

[42] Kwong W.K., Moran N.A. (2016). Gut microbial communities of social bees. Nat. Rev. Microbiol..

[43] Hasanin M.S., Abdelraof M., Hashem A.H., El Saied H. (2023). Sustainable bacterial cellulose production by Achromobacter using mango peel waste. Microb. Cell Fact..

[44] Manfredi A.P., Perotti N.I., Martínez M.A. (2015). Cellulose degrading bacteria isolated from industrial samples and the gut of native insects from Northwest of Argentina. J. Basic. Microbiol..

[45] Zhao Z.M., Liu Z.H., Zhang T., Meng R., Gong Z., Li Y., Hu J., Ragauskas A.J., Li B.Z., Yuan Y.J. (2023). Unleashing the capacity of *Rhodococcus* for converting lignin into lipids. Biotechnol. Adv..

[46] Roell G.W., Carr R.R., Campbell T., Shang Z., Henson W.R., Czajka J.J., Martín H.G., Zhang F., Foston M., Dantas G. (2019). A concerted systems biology analysis of phenol metabolism in *Rhodococcus opacus* PD630. Metab. Eng..

[47] Pérez-Cano F.J. (2022). Dietary Modulation of the Immune Function: Direct and Microbiota-Dependent Effect. Nutrients.

[48] Wang G., Wang Y.F., Li J.L., Peng R.J., Liang X.Y., Chen X.D., Jiang G.H., Shi J.F., Si-Ma Y.H., Xu S.Q. (2022). Mechanism of hyperproteinemia-induced blood cell homeostasis imbalance in an animal model. Zool. Res..

[49] Collins N., Belkaid Y. (2022). Control of immunity via nutritional interventions. Immunity.

[50] Batista K.K.S., Vieira C.S., Figueiredo M.B., Costa-Latgé S.G., Azambuja P., Genta F.A., Castro D.P. (2021). Influence of *Serratia marcescens* and *Rhodococcus rhodnii* on the Humoral Immunity of Rhodnius prolixus. Int. J. Mol. Sci..

[51] Song X., Zhang H., Zhang Y., Goh B., Bao B., Mello S.S., Sun X., Zheng W., Gazzaniga F.S., Wu M. (2023). Gut microbial fatty acid isomerization modulates intraepithelial T cells. Nature.

[52] Loo Y.T., Howell K., Chan M., Zhang P., Ng K. (2020). Modulation of the human gut microbiota by phenolics and phenolic fiber-rich foods. Compr. Rev. Food Sci. Food Saf..

[53] Morrison K.E., Jašarević E., Howard C.D., Bale T.L. (2020). It’s the fiber, not the fat: Significant effects of dietary challenge on the gut microbiome. Microbiome.

[54] Deleu S., Machiels K., Raes J., Verbeke K., Vermeire S. (2021). Short chain fatty acids and its producing organisms: An overlooked therapy for IBD?. EBioMedicine.

[55] He Y., Fu L., Li Y., Wang W., Gong M., Zhang J., Dong X., Huang J., Wang Q., Mackay C.R. (2021). Gut microbial metabolites facilitate anticancer therapy efficacy by modulating cytotoxic CD8 T cell immunity. Cell Metab..

[56] Yang W., Yu T., Huang X., Bilotta A.J., Xu L., Lu Y., Sun J., Pan F., Zhou J., Zhang W. (2020). Intestinal microbiota-derived short-chain fatty acids regulation of immune cell IL-22 production and gut immunity. Nat. Commun..

[57] Tao S., Wang J., Liu M., Sun F., Li B., Ye C. (2022). Haemolymph metabolomic differences in silkworms (*Bombyx mori* L.) under mulberry leaf and two artificial diet rearing methods. Arch. Insect Biochem. Physiol..

[58] Heng J., Liu H., Xu J., Huang X., Sun X., Yang R., Xia Q., Zhao P. (2022). KPI5 Is Involved in the Regulation of the Expression of Antibacterial Peptide Genes and Hemolymph Melanization in the Silkworm, *Bombyx mori*. Front. Immunol..

