# Supplementary material for: Effects of Habitual Dietary Change on the Gut Microbiota and Health of Silkworms
Source: Int J Mol Sci. 2024 Jan 31;25(3):1722. doi: 10.3390/ijms25031722 (PMC10855636; doi:10.3390/ijms25031722)
Supplement: Supplementary file 1 [file ijms-25-01722-s001.zip › ijms-2832548-supplementary.pdf]

Supplementary Material

Effects of Habitual Dietary Change on the Gut Microbiota and Health of Silkworms

Guang Wang [1,2,†], Xueyan Ding [1,2,†], Jiameng Yang [1,2], Lu Ma [1,2], Xiaoning Sun [1,2], Ruihong Zhu [1,2], Riming Lu [1,2], Zhitian Xiao [1,2], Zhiyi Xing [1,2], Jingbin Liu [1,2], Zhonghua Pan [1,2], Shiqing Xu [1,2] and Yanghu Sima [1,2,*]

[1] School of Biology and Basic Medical Sciences, Suzhou Medical College, Soochow University, Suzhou 215123, China; gwang1994@suda.edu.cn (G.W.); dingxueyan0120@163.com (X.D.); 20215221011@stu.suda.edu.cn (J.Y.); 20215221005@stu.suda.edu.cn (L.M.); xnsun@suda.edu.cn (X.S.); 20215221008@stu.suda.edu.cn (R.Z.); luriming1997@163.com (R.L.); 20225221012@stu.suda.edu.cn (Z.X.); 20225221013@stu.suda.edu.cn (Z.X.); 20225221003@stu.suda.edu.cn (J.L.); panzh@suda.edu.cn (Z.P.); szsqxu@suda.edu.cn (S.X.)
[2] Institute of Agricultural Biotechnology & Ecology (IABE), Soochow University, Suzhou 215123, China
* Correspondence: simyh@suda.edu.cn; Tel.: +86-512-6267-0538
[†] These authors contributed equally to this work.

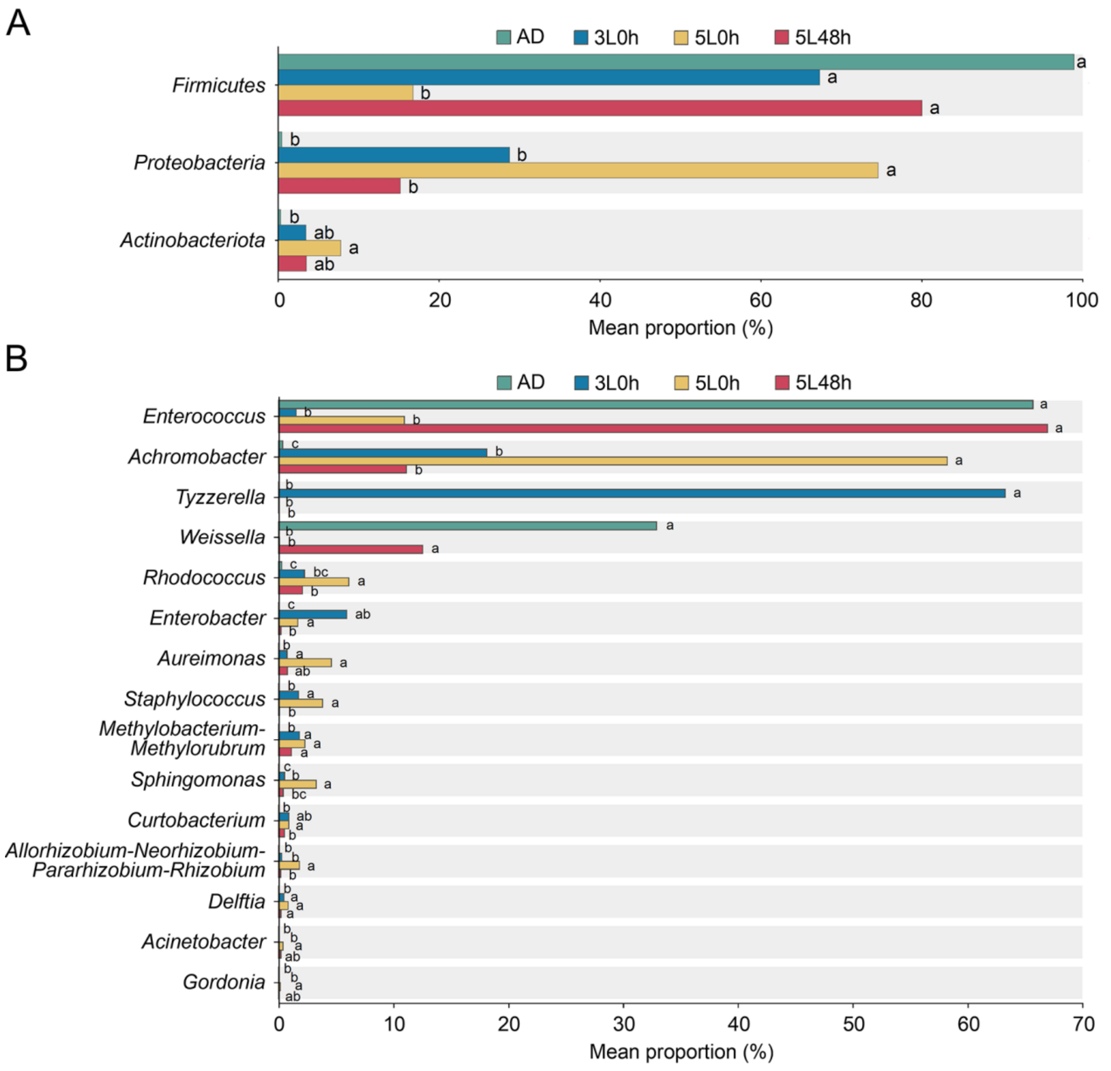

Figure S1. Differential analysis of microbial abundance in silkworms under different dietary patterns. (**A-B**) Species with abundance differences at the phylum level (**A**) and genus level (**B**) in the microbial communities of different groups. A *P* value < 0.05 indicated significant differences between groups, as shown with different letters.

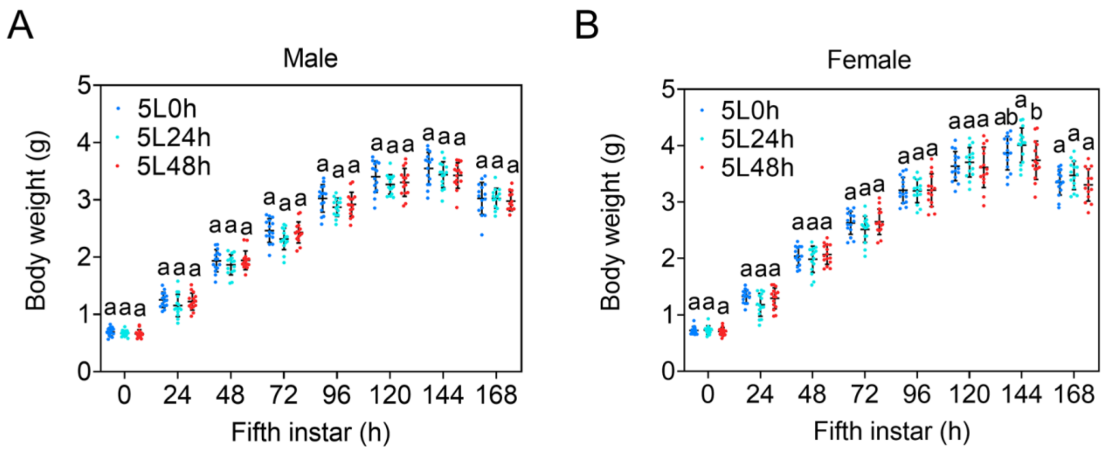

Figure S2. Differences in body weights of larvae with different dietary patterns. (**A-B**) Effects of different dietary transition timing during the fifth instar on the body weights of male and female larvae. $n = 15$. **A**, Male ($F = 0.534$; df = 14, 333; $P = 0.913$). **B**, Female ($F = 1.567$; df = 14, 335; $P = 0.087$). Two-way ANOVA. A P value < 0.05 indicated significant differences between groups, as shown with different letters.

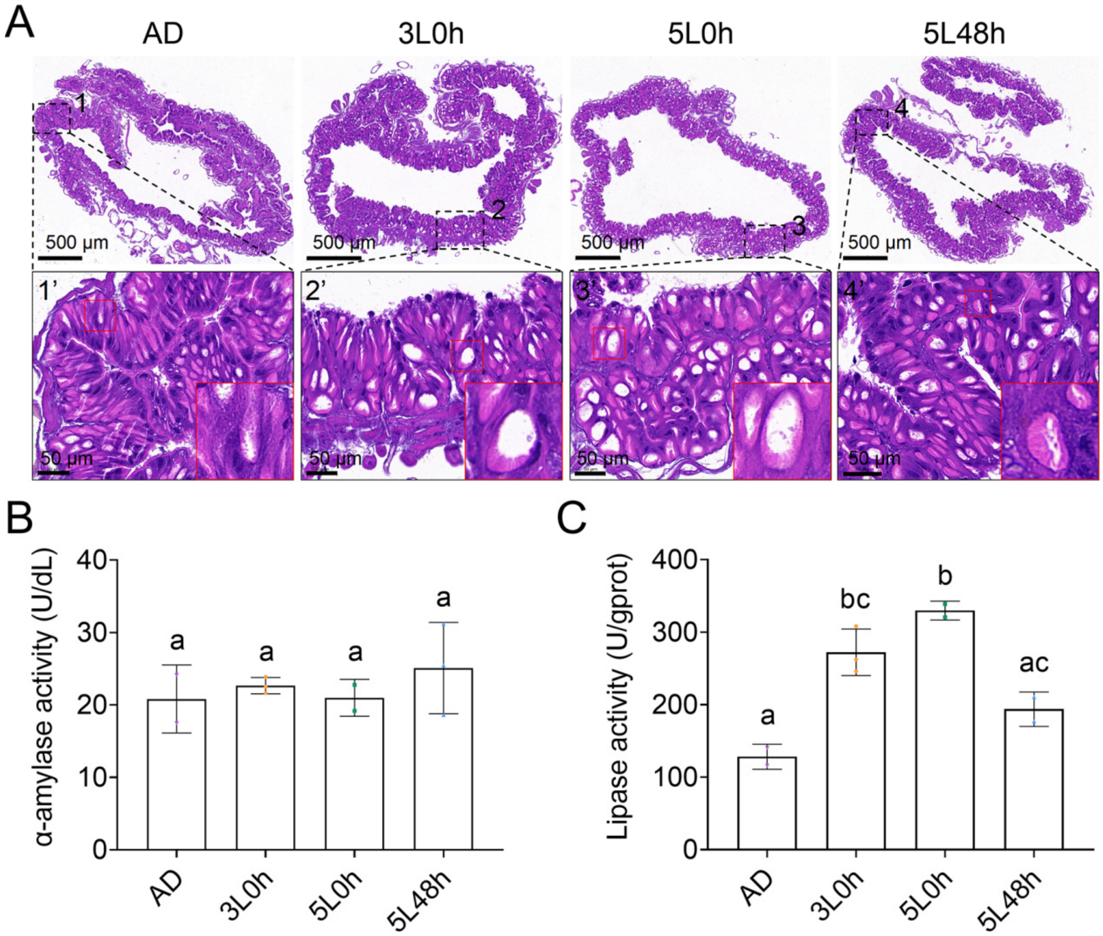

Figure S3. Differences in the digestion and absorption of larvae with different dietary patterns. (**A**) Hematoxylin–eosin staining image of midgut tissue sections. The midgut tissues came from silkworms at hour 72 of the fifth instar. 1, 2, 3 and 4 are partially enlarged images. (**B-C**) Digestive enzyme activity of intestinal fluid. $n = 3$, with ten individuals per group. **B**, α-amylase ($F = 0.547$; df = 3, 6; $P = 0.668$). **C**, Lipase ($F = 25.90$; df = 3, 5; $P = 0.002$). Two-way ANOVA. A P value < 0.05 indicated significant differences between groups, as shown with different letters.
